# Effects of a laughter program on body weight and mental health among Japanese people with metabolic syndrome risk factors: a randomized controlled trial

**DOI:** 10.1186/s12877-022-03038-y

**Published:** 2022-04-23

**Authors:** Narumi Funakubo, Eri Eguchi, Rie Hayashi, Mayumi Hirosaki, Kokoro Shirai, Kanako Okazaki, Hironori Nakano, Fumikazu Hayashi, Junichi Omata, Hironori Imano, Hiroyasu Iso, Tetsuya Ohira

**Affiliations:** 1 Department of Epidemiology, Fukushima Medical University School of Medicine, 1 Hikarigaoka, Fukushima, 960-1295 Japan; 2 Public Health, Department of Social Medicine, Osaka University Graduate School of Medicine, Osaka, Japan; 3 Center for Southeast Asian Studies, Kyoto University, Kyoto, Japan; 4 Department of Physical Therapy, Fukushima Medical University School of Health Sciences, Fukushima, Japan; 5 Radiation Medical Science Center for the Fukushima Health Management Survey, Fukushima Medical University, Fukushima, Japan; 6 Department of Anatomy, Fukushima Medical University School of Medicine, Fukushima, Japan; 7 Department of Public Health, Kindai University Faculty of Medicine, Osaka, Japan

**Keywords:** Body mass index, Happiness, Health-related quality of life, Laughter yoga, Revised life orientation test, Stress

## Abstract

**Background:**

While there have been several intervention studies on the psychological effects of laughter, few have examined both the psychological and physical effects. This study investigates the effects of a laughter program on body weight, body mass index (BMI), subjective stress, depression, and health-related quality of life (HRQOL) among Japanese community-dwelling individuals using a randomized controlled trial with a waitlist.

**Methods:**

Overall, 235 participants (37 men and 198 women) aged 43–79 years (mean 66.9, median 67.0) were randomized into laughter intervention and control groups (*n* = 117 and *n* = 118, respectively) to participate in a 12-week laughter program. Body weight, subjective stress, subjective well-being, and HRQOL were measured at the baseline, with a 12-week follow-up. The laughter program intervention’s effects on these factors were analyzed using an analysis of covariance adjusted by age, sex, risk factors, medication, and area. Furthermore, Pearson’s correlation and a general linear model analyzed the relationship between participants’ BMI and psychological index changes.

**Results:**

The comprehensive laughter program significantly improved the mean body weight (*p* = 0.008), BMI (*p* = 0.006), subjective stress (*p* = 0.004), subjective well-being (*p* = 0.002), optimism (*p* = 0.03), and physical component summary (PCS) scores of HRQOL (*p* = 0.04). A similar tendency occurred for the mean changes in BMI and subjective stress score by area, sex, and age. Moreover, there was a significant and negative correlation between the change in BMI and PCS change (*p* = 0.04).

**Conclusion:**

The comprehensive 12-week laughter intervention program, mainly comprising laughter yoga, significantly improved physical and psychological functions such as body weight, BMI, subjective stress, subjective well-being, and HRQOL among predominantly elderly Japanese community-dwelling individuals with metabolic syndrome risk factors. Moreover, PCS improved among participants who reduced BMI after the intervention. These results suggest that the laughter program may help reduce body weight in participants with metabolic syndrome risk factors by reducing stress and improving HRQOL and mental health factors, such as subjective well-being and optimism.

**Trial registration:**

Registered with the University Hospital Medical Information Network Clinical Trials Registry UMIN-CTR000027145 on 27/04/2017.

**Supplementary Information:**

The online version contains supplementary material available at 10.1186/s12877-022-03038-y.

## Background

Metabolic syndrome is a combination of (visceral) obesity, hypertension, diabetes, and dyslipidemia that can easily lead to cerebrovascular and heart diseases. The relationship between metabolic syndrome and lifestyle factors such as diet and exercise is well known; however, in recent years, it has become clear that psychosocial stresses, such as chronic work stress, can also affect metabolic syndrome development [1, 2]. Therefore, a decrease in psychological stress may improve metabolic syndrome and reduce the risk of death from cerebrovascular disease and heart disease.

Whereas, since Norman Cousins’s 1976 report on the effects of laughter on pain relief [3], the effects of laughter have received more attention, and extensive research has been conducted regarding the links between laughter and health and laughter and diseases. Recently, cross-sectional studies and prospective cohort studies have reported that, on rare occasions, low frequency of laughter in daily life increased the risk of death [4] and needing care [5]. Ikeda et al. also reported an association between the frequency of laughter and hypertension, which are risk factors for cardiovascular diseases [6]. In a large prospective study, we also have reported that enjoying life significantly reduces the risk of cardiovascular death [7] and that people who laugh less than daily have a 1.21-fold higher risk of suffering heart disease than those who laugh daily [8]. Thus, laughter may be involved not only in lifespan but also in extending healthy life expectancy.

Two laughter interventions have had some success in reducing illness and symptoms [9]: spontaneous laughter interventions, which involve watching comedy shows, clown performances, *rakugo* (a traditional Japanese comic story) and *manzai* (a Japanese comic dialog); and simulated laughter interventions, which involve laughter and exercise, such as laughter yoga. Spontaneous laughter studies found that watching comic videos activated natural killer cells in 10 type 2 diabetic patients [10], laughing for 3 h at a variety theater, such as *manzai*, activated natural killer cells and enhanced immunity [11]. Spontaneous laughter from watching *rakugo* decreased IL-6 and improved symptoms in 26 rheumatoid arthritis patients [12]. Dr. Madan Kataria in India started laughter yoga in 1995, and it is spreading quickly around the world. There are currently over 10,000 laughter yoga clubs in more than 100 countries including Japan. Laughter yoga has reportedly been effective in treating depression among the elderly [13], in reducing stress before chemotherapy in cancer patients [14], and improving symptoms of irritable bowel syndrome [15], among other mental and physical effects. Therefore, laughter yoga could reduce stress and prevent cardiovascular disease. Laughter yoga’s combination of laughter and exercise is reportedly effective and highly sustainable, even with light exercise [16]. Based on the above, a laughter program combining laughter yoga (simulated laughter) and *rakugo* (spontaneous laughter) should be effective for both physical and mental health. However, no study has examined a laughter program’s physical and psychological effects using a randomized controlled trial (RCT) in a comprehensive and large group setting. The purpose of this intervention study was to compare the effects of the comprehensive laughter program on physical and psychological health indicators and the relationship between these two among Japanese community-dwelling individuals at risk of metabolic syndrome by using an RCT design.

## Methods

### Study design

This study was an RCT with waitlist control held in Fukushima, Osaka, and Okayama, with an allocation ratio of 1:1. The follow-up examination was conducted after 12 weeks for both groups.

### Participants

Men and women ages 40 to 79 who had one or more risk factors of metabolic syndrome [17] such as higher obesity grade, hypertension (systolic blood pressure [SBP] ≥130 mmHg, diastolic BP [DBP] ≥85 mmHg), hemoglobin A1c [HbA1c] ≥5.6%, high-density lipoprotein cholesterol < 40 mg/dL, or triglyceride ≥150 mg/dL, or were taking medication for hypertension, diabetes, or cholesterol, and who lived in or near Fukushima, Fukushima Prefecture; Osaka, Osaka Prefecture; or Okayama, Okayama Prefecture, Japan, were eligible for inclusion in this study. Participants were excluded if they were under treatment for severe cardiovascular disease or severe stroke. Both SBP and DBP were measured twice using an automatic sphygmomanometer, and the mean value of the two measurements was used to determine the presence of hypertension. HbA1c was measured using a cobas b 101 plus HbA1c analyzer (Roche Diagnostics K.K., Tokyo, Japan).

The studies were conducted at Fukushima Medical University in Fukushima, Osaka University in Osaka, and Okayama University in Okayama, Japan.

### Interventions

The laughter program was composed of an approximately 30-min lecture about laughter, health, and diseases, or an approximately 30-min appreciation of *rakugo* by a professional performer of *rakugo*, a traditional form of Japanese comic storytelling, and an approximately 60-min laughter yoga class by the laughter yoga teachers in the Japan Laughter Yoga Association. *Rakugo* was regarded as part of the laughter lectures, and two lectures were given. *Rakugo* lectures were designed to make the participants laugh simultaneously as learning about laughter and health. The timetable of the laughter program is shown in Table 1. The intervention classes with the laughter groups were conducted between June and September 2017 and between May and July 2018 in Fukushima, November 2017 and February 2018 in Osaka, and May and July 2017 in Okayama. Participants attended the program 8 times in Fukushima and Osaka and 10 times in Okayama for the approximately 12-week intervention period, and their attendance was recorded. Although the number of intervention classes by area was different, the program’s structure was the same. During the waiting period, the control group went about their daily lives as usual. However, intervention classes were conducted after the control period. Laughter group participants were asked to keep a laughter diary during the intervention period to monitor their laughter frequency outside the program.

**Table 1:** Timetable of the laughter program

Timetable (min)	Laughter program (8–10 times)
0–5	Instruction
5–30	Lecture or *Rakugo* (2 times)
30–85	Laughter yoga
30–35	Initial instruction
35–55	Perform 10 basic laughter exercises
55–60	Rest
60–75	Exercise: various kinds of laughter yoga exercise
75–85	Breathing
85–90	Closing

### Measurements

#### Body weight and abdominal circumference

Body weight was measured using a UC-322 (A&D Co. Ltd., Tokyo, Japan) in Fukushima and Osaka and a WB-260A (Tanita, Tokyo, Japan) in Okayama. Height was self-reported, and body mass index (BMI) was calculated as weight/height (kg/m^2^). Abdominal circumference was measured halfway between the lower border of the ribs and iliac crest using a measuring tape.

#### Self-administered questionnaires

Participants answered questions about lifestyle and behaviors such as habitual alcohol intake, smoking status, habitual exercise, frequency of laughter, subjective mental stress, subjective well-being, health-related QOL (HRQOL), depressive symptoms, and optimism. The habitual exercise was defined as having physical activity at least twice a week through exercise or recreation and was evaluated by the presence or absence of such activity. The frequency of laughter was evaluated by whether or not participants laughed almost daily. Subjective mental stress was evaluated by asking, “What is the level of stress in your daily life?” using a 4-point scale (1: low, 2: medium, 3: high, 4: extremely high). Subjective well-being was evaluated by asking, “What is the level of your happiness?” using a score ranging from 1 to 10, and the higher scores indicate greater happiness.

The HRQOL was evaluated using the Japanese version of the 8-Item Short-Form Health Survey (SF-8) [18, 19]. The SF-8 includes 8 questions on a 5- or 6-point scale, evaluating the following 8 domains: physical functioning, role physical, bodily pain, general health perception, vitality, social functioning, role emotional, and mental health. The point scales are normalized using Japanese standards, indicating a score of 50 points that is the Japanese average, and higher scores indicate a better QOL. The first four domains (physical functioning, role physical, bodily pain, and general health perception) indicate the physical component summary (PCS), while the latter four domains (vitality, social functioning, role emotional, and mental health) indicate the mental component summary (MCS).

Depressive symptoms were evaluated using the Japanese version of the Geriatric Depression Scale 15 (GDS-15-J) [20, 21]. The GDS-15-J comprises15 questions, with scores ranging from 0 to 15, with and higher scores indicating more depressive tendencies [22].

Optimism was evaluated through the Japanese version of the revised Life Orientation Test (LOT-R) [23, 24]. The LOT-R includes 10 items, of which three measure optimism, 3 measure pessimism, and 4 act as fillers. Each item uses a 5-point scale (4: strongly agree, 3: agree, 2: neutral, 1: disagree, 0: strongly disagree) [23]; a higher score indicates more optimism.

### Sample size

The power calculation based on a previous elderly person study, which found that mean body weight decreased by 0.63 kg after a laughter and exercise program intervention [16], were used to determine the sample size. While a 0.63 kg weight reduction in an individual might not have clinical significance, but when considered as population data, it could be clinically significant because the positive association between weight variability and cardiovascular disease was reported in the 5209 individuals in the Framingham cohort [25] was considered relatively important. Therefore, under an assumption of a body weight decrease of 0.63 kg with a standard deviation of 1.52, a two-sided significance level of 0.05, and a statistical power of 80%, the total sample size was determined to be 186, and 93 each in the intervention and control groups. When a possible dropout rate of 10% was factored in, 206 participants were required as the suitable sample size for this study.

### Randomization

After the baseline examination, participants were randomly divided into a laughter group and a control group (waitlist) in each area stratified by sex, age (< 65 years old and ≥ 65 years old), and overweight status (body mass index [BMI] ≥25 kg/m^2^ or waist circumference ≥ 85 cm for men, ≥90 cm for women according to definition and diagnostic criteria for metabolic syndrome in Japan [17]). After stratifying the participants by sex and determining whether they were overweight or not, two groups were assigned by administrative staff in a 1:1 ratio to each area. The assignment was made using Excel with a random number ranging from 0 to 1, with 0.5 as the cutoff value.

### Statistical analysis

The intervention program was held in Fukushima, Osaka, and Okayama. Even though the same program was used in each area, because the instructors were different, a pooled analysis was conducted to determine if the results in each area were the same trend. Mean values and ratios of the baseline characteristics for the laughter group (*n* = 117) and the control group (*n* = 118) were calculated. Normality tests were conducted, the normally distributed results were compared using an unpaired t-test. A Mann-Whitney U test was used to compare the non-normally distributed results, and a chi-square test was used to determine the ratios. The intervention effects of the laughter program on body weight, BMI, abdominal circumference, subjective mental stress, subjective well-being, GDS-15-J, LOT-R, and HRQOL were analyzed using an analysis of covariance adjusted by age, sex, metabolic syndrome risk factors, medication, and area, and each dependent variable value at baseline. To evaluate the effects of the laughter program on the physical and psychological status, we analyzed the relationship between the changes in BMI and psychological index in the laughter group using Pearson’s correlation and showed graphically using a general linear model. An intention to treat analysis was adopted. As there were no significant differences in the number of dropouts in both groups, primarily because of scheduling conflicts, these were considered random missing values that were unrelated to the intervention. That is, if there were any missing values in the analyzed individuals, the missing values were substituted with the baseline values for the analyses. The sensitivity analysis results without the missing value substitutions are shown in Table S1 in the Supplemental Information. SAS statistical software version 9.4 (SAS Institute Inc., Cary, NC, USA) was used for analyzing the data. *P*-values of less than 0.05 were considered significant.

## Results

### Characteristics of participants at baseline

**Fig. 1:**
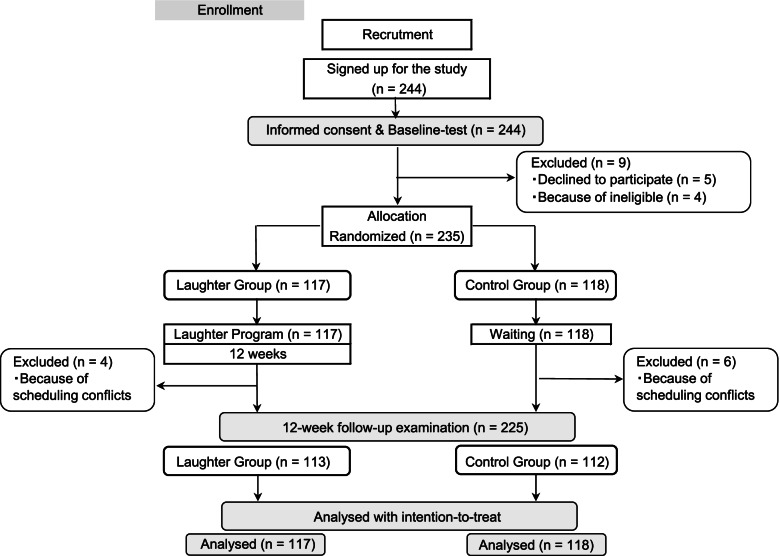
Flowchart and schedule of trial

Two hundred thirty-five participants (37 men and 198 women) aged 43 to 79 years were eligible for inclusion. The flowchart in Fig. 1 shows that these 235 participants were randomized into the laughter and control groups (*n* = 117 and *n* = 118, respectively). Additionally, 9 women and one man dropped out before the 12-week follow-up examination because of scheduling conflicts (*n* = 113 and *n* = 112, respectively). Participants were recruited in Fukushima between 2017 and 2018 and in Osaka and Okayama in 2017 through flyers and newspaper advertisements. Participant characteristics at baseline are shown in Table 2. Participants were predominantly female (84.3%) and older than 60 years old (80.4%). There were no differences between the laughter and control groups at baseline. Of the 235 participants, 95 were in Fukushima, 62 were in Osaka, and 78 were in Okayama, and the average attendance rate was more than 84.8%.

**Table 2:** Baseline characteristics of participants

Baseline variables	Laughter Group (*n* = 117)	Control Group (*n* = 118)	*P* value	Total (*n* = 235)
	Mean ± SD	Mean ± SD		Mean ± SD
Age, years	66.8 ± 7.2	67.1 ± 8.8	0.38	66.9 ± 8.0
Women, %	98 (83.8)	100 (84.8)	0.84	198 (84.3)
Body Mass Index, kg/m^2^	23.1 ± 3.5	22.8 ± 3.2	0.35	23.0 ± 3.3
Abdominal circumference, cm	85.4 ± 10.0	85.0 ± 10.0	0.78	85.2 ± 10.0
Obesity^a^, %	47 (40.2)	45 (38.1)	0.75	92 (39.2)
Diabetics^b^, %	104 (88.9)	109 (92.4)	0.36	213 (90.6)
Hypertension^c^, %	80 (68.4)	82 (69.5)	0.85	162 (68.9)
Dyslipidemia^d^, %	68 (58.1)	64 (54.2)	0.55	132 (56.2)
Habitual alcohol intake, %	38 (32.5)	41 (34.8)	0.71	79 (33.6)
Smoking status, %	2 (1.7)	1 (0.85)	0.56	3 (1.3)
Habitual exercise^e^, %	77 (65.8)	75 (63.6)	0.72	152 (64.7)
The frequency of laughter^f^, %	34 (29.1)	23 (19.5)	0.09	57 (24.3)
Subjective stress, score	2.3 ± 0.9	2.2 ± 0.8	0.41	2.2 ± 0.8
subjective well-being, score	7.6 ± 1.7	7.5 ± 1.4	0.29	7.6 ± 1.5
Geriatric depression scale 15, score	3.7 ± 3.0	4.1 ± 3.1	0.49	3.9 ± 3.1
The Revised Life Orientation Test, score	13.4 ± 3.3	13.0 ± 3.2	0.45	13.2 ± 3.3
SF-8 Health Survey Scoring, score				
Physical component summary	46.6 ± 7.8	46.8 ± 7.6	0.99	46.7 ± 7.7
Mental component summary	48.8 ± 6.2	49.3 ± 6.7	0.30	49.0 ± 6.5
Physical functioning	47.4 ± 8.0	47.5 ± 8.1	0.75	47.5 ± 8.0
Role physical	48.1 ± 8.0	48.8 ± 6.7	0.90	48.5 ± 7.3
Bodily pain	48.2 ± 8.1	48.5 ± 8.9	0.69	48.3 ± 8.5
General health perception	48.4 ± 6.4	48.5 ± 7.1	0.85	48.5 ± 6.8
Vitality	49.2 ± 6.0	49.5 ± 5.9	0.68	49.4 ± 5.9
Social functioning	48.3 ± 8.3	48.7 ± 8.8	0.47	48.5 ± 8.5
Role emotional	49.2 ± 6.4	49.4 ± 6.2	0.76	49.3 ± 6.3
Mental health	48.7 ± 6.5	49.3 ± 6.9	0.38	49.0 ± 6.7

Values are means ± standard deviation (SD) and numbers (ratios). *P* values indicate the significance of the differences between the Laughter and Control groups. Mean values and ratios of the baseline characteristics for the laughter and the control groups were calculated and compared using an unpaired t-test, Mann-Whitney U test or chi-square test

^a^Obesity: higher body mass index (≥25 kg/m^2^) or higher waist circumference (≥85 cm for men, ≥90 cm for women)

^b^Diabetics: HbA1c ≥5.6% or with medication

^c^Hypertension: systolic blood pressure ≥ 130 mmHg, diastolic blood pressure ≥ 80 mmHg or with medication

^d^Dyslipidemia: high-density lipoprotein cholesterol < 40 mg/dL, triglyceride ≥150 mg/dL or with medication

^e^Habitual exercise: the frequency of people who have physical activity at least twice a week by exercise or recreation

^f^The frequency of laughter: the frequency of people who laugh daily. SF-8: 8-Item Short Form Health Survey

### Body weight, abdominal circumference, and mental health

The changes in variables at the baseline, 12-week follow-up examinations in both the laughter and control groups, and the intervention effects of the laughter program are shown in Table 3. The intervention effects on body weight and BMI were found significantly in 2 groups (*p* = 0.008, *p* = 0.006, respectively). There was no significant decrease in abdominal circumference. There were significant improvements in the subjective mental stress scores (*p* = 0.004), in the LOT-R scores (*p* = 0.03), and in the subjective well-being scores (*p* = 0.002). Furthermore, there were significant increases in the PCS (*p* = 0.04), physical functioning (*p* = 0.02), role physical (*p* = 0.03) and mental health scores (*p* = 0.02). Furthermore, the changes in bodily pain, general health perception, vitality, and role emotional tended to increase (*p* = 0.09, *p* = 0.09, *p* = 0.07, and *p* = 0.06, respectively). There were no significant increases in the social functioning and MCS scores of HRQOL. As a sensitivity analysis, a similar analysis was conducted by excluding those who attended fewer than half the sessions (*n* = 4) in the laughter group, and the results were similar.

**Table 3:** Changes and intervention effects in physical and mental health scores at baseline and follow-up examinations

Variables	Group	Examination ^a^		Changes	Changes between laughter and control groups ^b^	95% confidence intervals ^c^	Multivariable-adjusted changes	*P* value ^d^
		Baseline	12-week follow-up					
Number	Laughter	117	117					
	Control	118	118					
Body mass index, kg/m^2^	Laughter	23.1 ± 3.5	22.9 ± 3.3	−0.27 ± 0.5	− 0.19 ± 0.49	− 0.31 – − 0.06	− 0.27	0.006
	Control	22.8 ± 3.2	22.7 ± 3.1	− 0.09 ± 0.5			− 0.09	
Body weight, kg	Laughter	56.7 ± 10.4	55.9 ± 10.3	−0.65 ± 1.2	−0.43 ± 1.19	− 0.73 – − 0.12	− 0.64	0.008
	Control	56.0 ± 9.4	55.6 ± 9.2	− 0.23 ± 1.2			− 0.24	
Abdominal circumference, cm	Laughter	85.4 ± 10.0	85.3 ± 9.8	−0.09 ± 3.2	−0.01 ± 3.42	−0.89 – 0.87	− 0.06	0.92
	Control	85.0 ± 10.0	84.9 ± 9.7	−0.08 ± 3.6			− 0.11	
Subjective mental stress, score	Laughter	2.3 ± 0.9	2.1 ± 0.8	− 0.18 ± 0.6	−0.24 ± 0.61	−0.40 – − 0.08	−0.17	0.004
	Control	2.2 ± 0.8	2.2 ± 0.8	0.06 ± 0.6			0.05	
Subjective well-being, score	Laughter	7.6 ± 1.7	8.0 ± 1.4	0.40 ± 1.3	0.39 ± 1.20	0.08–0.70	0.42	0.002
	Control	7.5 ± 1.4	7.6 ± 1.5	0.01 ± 1.1			−0.01	
Geriatric depression scale 15, score	Laughter	3.7 ± 3.0	3.2 ± 2.9	−0.55 ± 2.0	−0.28 ± 2.11	−0.83 – 0.26	− 0.60	0.14
	Control	4.1 ± 3.1	3.8 ± 3.2	−0.27 ± 2.2			− 0.22	
The Revised Life Orientation Test, score	Laughter	13.4 ± 3.3	14.4 ± 3.4	0.92 ± 2.5	0.56 ± 2.57	−0.10 – 1.22	0.99	0.03
	Control	13.0 ± 3.2	13.3 ± 3.4	0.36 ± 2.6			0.30	
SF-8 Health Survey Scoring, score								
Physical component summary	Laughter	46.6 ± 7.8	48.5 ± 6.7	1.98 ± 7.6	1.88 ± 7.59	− 0.08 – 3.83	1.94	0.04
	Control	46.8 ± 7.6	46.9 ± 8.3	0.10 ± 7.6			0.14	
Mental component summary	Laughter	48.8 ± 6.2	49.8 ± 6.5	1.01 ± 6.5	1.12 ± 6.31	−0.50 – 2.74	0.91	0.22
	Control	49.3 ± 6.7	49.1 ± 7.3	−0.11 ± 6.1			− 0.01	
Physical functioning	Laughter	47.4 ± 8.0	49.2 ± 6.8	1.78 ± 7.8	2.22 ± 8.35	0.08–4.37	1.75	0.02
	Control	47.5 ± 8.1	47.1 ± 9.3	− 0.44 ± 8.8			− 0.42	
Role physical	Laughter	48.1 ± 8.0	49.6 ± 6.2	1.52 ± 7.1	2.17 ± 7.35	0.28–4.06	1.33	0.03
	Control	48.8 ± 6.7	48.1 ± 7.6	− 0.65 ± 7.6			−0.47	
Bodily pain	Laughter	48.2 ± 8.1	50.5 ± 8.2	2.26 ± 9.2	1.67 ± 8.51	− 0.52 – 3.85	2.25	0.09
	Control	48.5 ± 8.9	49.1 ± 8.7	0.59 ± 7.8			0.61	
General health perception	Laughter	48.4 ± 6.4	50.6 ± 6.2	2.22 ± 5.9	1.25 ± 6.04	−0.31 – 2.80	2.20	0.09
	Control	48.5 ± 7.1	49.5 ± 7.3	0.97 ± 6.2			1.00	
Vitality	Laughter	49.2 ± 6.0	50.7 ± 6.3	1.47 ± 6.3	1.39 ± 5.78	−0.10 – 2.87	1.41	0.07
	Control	49.5 ± 5.9	49.6 ± 6.3	0.09 ± 5.2			0.15	
Social functioning	Laughter	48.3 ± 8.3	49.5 ± 8.1	1.20 ± 8.9	0.91 ± 8.40	−1.25 – 3.07	1.07	0.48
	Control	48.7 ± 8.8	49.0 ± 8.1	0.29 ± 7.9			0.42	
Role emotional	Laughter	49.2 ± 6.4	50.2 ± 5.3	0.99 ± 6.5	1.55 ± 6.60	− 0.15 – 3.25	0.93	0.06
	Control	49.4 ± 6.2	48.9 ± 7.7	−0.55 ± 6.7			−0.49	
Mental health	Laughter	48.7 ± 6.5	50.5 ± 6.4	1.78 ± 6.0	1.86 ± 6.12	0.29–3.43	1.66	0.02
	Control	49.3 ± 6.9	49.3 ± 7.0	−0.08 ± 6.2			0.04	

Data are represented as means ± standard deviation and mean change. *P* values indicate the intervention effects of changes in the intervention and non-intervention periods. Changes in each group are shown as the value of the 12-week follow-up examination minus the value of baseline examination. The intervention effects of the laughter program on variables were analyzed using an analysis of covariance adjusted by age, sex, metabolic syndrome risk factors, medication, and area, and each dependent variable value at baseline

^a^Values are mean ± standard deviation

^b^Baseline and 12-week follow-up changes between the laughter and control groups

^c^95% confidence intervals between changes in baseline and 12-week follow-up of between the laughter and control groups

^d^*P* values for comparing the adjusted changes from baseline to 12-week follow-up in the laughter and control groups using an analysis of covariance adjusted by age, sex, medication and area, and each dependent variable value at baseline. SF-8: 8-Item Short Form Health Survey

### Forest plots for pooled analyses

**Fig. 2:**
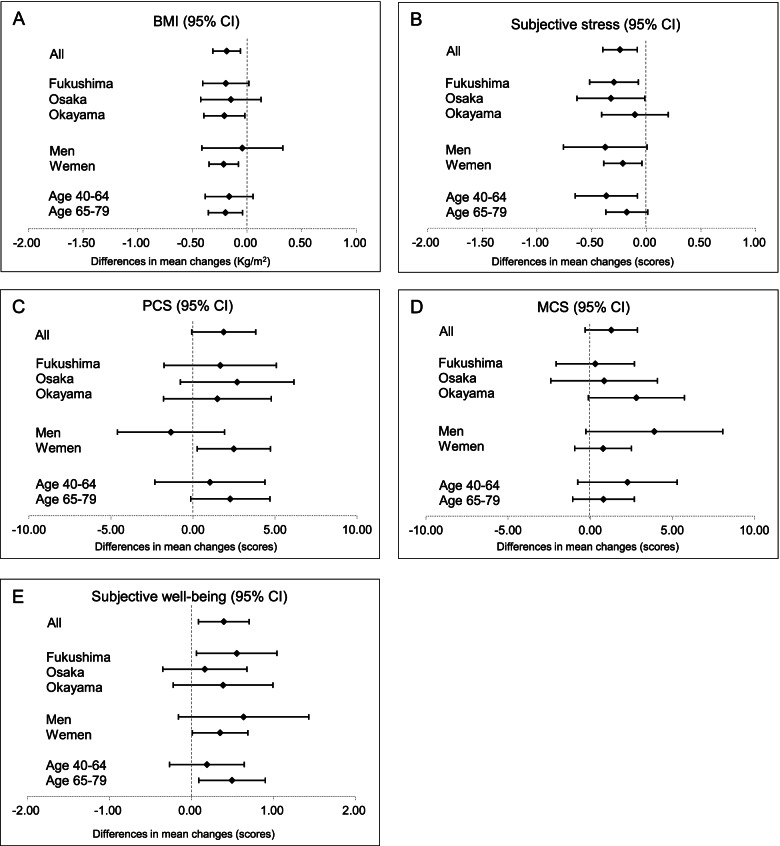
Forest plots, with means shown by closed circles and whiskers representing the 95% confidence interval. **A** The differences in the mean changes in body mass index (BMI) from baseline to 12-week follow-up between the laughter and control groups by area, sex, and age. A lower score indicates better BMI. **B** The differences in the mean changes in subjective stress from baseline to 12-week follow-up between the laughter and control groups by area, sex, and age. A lower score indicates lower subjective stress. **C** The differences in the mean changes in physical component summary (PCS) from baseline to 12-week follow-up between the laughter and control groups by area, sex, and age. A higher score indicates better PCS. **D** The differences in the mean changes in Mental component summary (MCS) from baseline to 12-week follow-up between the laughter and control groups by area, sex, and age. A higher score indicates better MCS. **E** The differences in the mean changes in subjective well-being from baseline to 12-week follow-up between the laughter and control groups by area, sex, and age. A higher score indicates better subjective well-being. CI: confidence interval

Forest plots of the differences in the mean changes in BMI, subjective stress, PCS, MCS, and subjective well-being scores from baseline to 12-week follow-up between laughter and control groups by area, sex, and age are shown in Fig. 2. A similarly decreasing tendency for BMI and subjective stress score was observed by area, sex, and age (Fig. 2A, B, respectively). Area, women and age observed a similarly increasing tendency for PCS scores (Fig. 2C). Further, a similarly increasing tendency for MCS and subjective well-being score was observed by area, sex, and age (Fig. 2D and E, respectively).

### Relationship between the changes in BMI and PCS scores

**Fig. 3:**
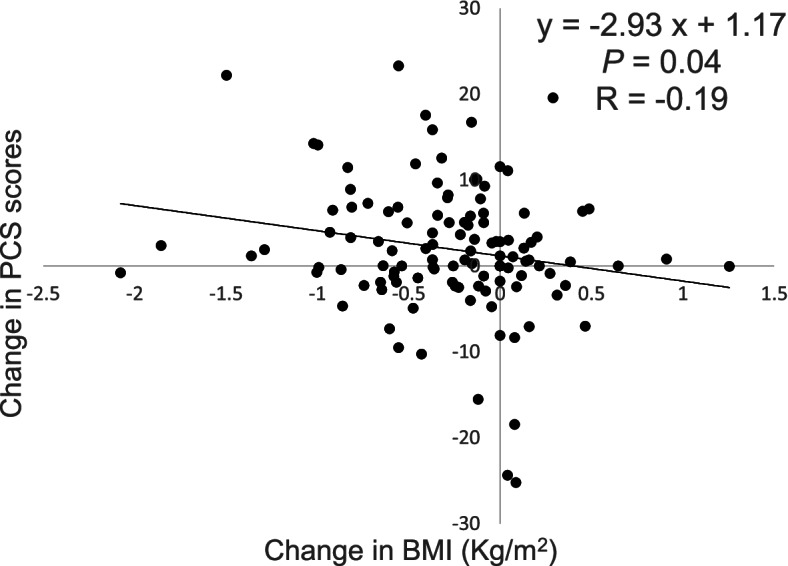
The relationship between the changes in BMI and PCS scores in the laughter group. The change in body mass index (BMI) had a negative correlation with the change in physical component summary (PCS) scores in participants after the intervention (*n* = 117, *p* = 0.04)

We analyzed the associations between BMI and psychological index changes such as subjective stress, HRQOL, and subjective well-being in the laughter group. There was a significant and negative correlation between BMI change and PCS change (*r* = − 0.19, *p* = 0.04), but there were no significant correlations in the other index. The relationship between the changes in BMI and PCS scores in the laughter group is shown in Fig. 3.

No harm or side effects occurred throughout the trial.

## Discussion

The present study indicated that a comprehensive laughter program of 8–10 classes for 12 weeks significantly improved physical and psychological function such as body weight, BMI, subjective stress, subjective well-being, and HRQOL among Japanese community-dwelling individuals with metabolic syndrome risk factors. Additionally, participants who reduced BMI after intervention improved PCS scores, suggesting that the improvement of HRQOL and obesity are in a mutualistic relationship.

Stress can trigger or aggravate many diseases and conditions [26]. The weight loss experienced in the laughter group could be attributable to both the exercise and the stress-reducing effects of the laughter and the breathing techniques. Genuine voiced laughter has been identified as an aerobic exercise. According to Buchowski et al., genuine voiced laughter causes a 10–20% increase in energy expenditure and heart rate above resting values, and 10–15 min of daily laughter being found to increase total energy expenditure by 10–40 kcal [27]. The 2011 Physical Activity Compendium reported that the Metabolic Equivalent for Task (METs) value for standing was 1.8 [28] and that for laughter was at least 2 METs. Therefore, as laughter yoga includes both laughter and light exercise, it was assumed to be at least equivalent to 2–3 METs of exercise. Increasing laughter by watching *rakugo* significantly decreased cortisol, a stress hormone [29]. Furthermore, the frequency of laughter decreased under stress [30], and there was an association between eating disorders, such as overeating and psychological distress [31]. Tanaka et al. also reported that abdominal breathing techniques such as yoga relieved stress among 14 elderly persons by reducing the pressure rate product, an index of myocardial oxygen consumption [32], and enhanced parasympathetic nervous activities, such as decreases in blood pressure and heart rate. These indicate that laughing is a light aerobic exercise and a form of abdominal breathing that reduces stress and attenuates sympathetic nervous activities. Thus, laughter can affect the metabolic syndrome through the endocrine system such as a hypothalamic-pituitary-adrenocortical system, potentially reducing stress-induced overeating and leading to weight loss.

Furthermore, the laughter program intervention may have brought about positive thinking and reduced participants’ stress levels. Some studies have shown a relation between positive thinking, optimism, and subjective well-being [33, 34]. For instance, optimists perceived greater upward orientation when faced with a difficult situation [35]. In our study, after the intervention, average subjective well-being, optimistic measure, and mental health and role emotional scores of HRQOL improved, supporting results from previous studies [33–35]. The pessimistic group with lower-than-median optimism scores also showed significant improvements after the intervention, including weight loss and increases in scores of HRQOL and subjective well-being. The effects of the laughter intervention were similar (data not shown). Moreover, there were tendencies of positive correlations between the changes in subjective well-being and role emotional scores and between the changes in future optimism and mental health. Furthermore, there were negative correlations between the changes in subjective stress, mental health, and role emotional scores. Besides, there are various poses that use parts or the whole of the body in laughter yoga. The effect of laughter yoga as an exercise may have improved participants’ physical function by increasing the amount of exercise and the body’s flexibility more than usual. These results support this improvement in the scores of physical function and role physical in HRQOL in this study.

Furthermore, because social networks (e.g., the frequency of meeting people) increased significantly in the laughter group in our study (data not shown), participation in a laughter program may improve social functioning. Some meta-analytic studies of the effects of laughter report it is beneficial for the body and mind [9, 36, 37]. In daily life, factors that increase laughter are related to participation in social activities and creating opportunities to meet with people, such as family and friends [38]. Therefore, increased social activity is an important factor in extending healthy life expectancy. This intervention may have created opportunities for more social activities, such as opportunities for participants to get to know each other, communication, and accompany each other to other classes and events.

Through body composition changes and muscle strength, upper body muscle training increased physical and mental QOL in breast cancer survivors [39]. Thus, the inverse association between the changes in BMI and PCS in the laughter group suggests that the laughter program may have improved PCS through weight loss by body composition changes and increased muscle mass. It is also possible that the increase in PCS allowed participants to become more active, resulting in a decrease in BMI. These causal relationships need to be continually investigated. Also, in this study, weight, BMI, abdominal circumference, and HRQOL have improved significantly in obese participants in the intervention group compared to non-obese participants (data not shown). These results suggest that it may effectively delay and prevent the progression to metabolic syndrome.

Recently, it was reported that both natural (spontaneous) and fake (simulated) laughter activated the same active brain regions [40], indicating the importance of the behavior of laughter. Furthermore, a meta-analysis of laughter reported that laughter without humor was more effective in improving depressive symptoms [9], suggesting that it is important to increase the opportunity for laughter, regardless of the means used. In our study, laughter yoga consists only of laughing vocalizations and actions and allows laughter to be performed without humor and without something being funny. In fact, laughter behaviors, which include the laughter yoga, in the Fukushima intervention group participants increased by an average of 3 h/week compared to the control group (data not shown). Furthermore, laughter yoga is cost-effective because it is inexpensive and can be easily practiced by an individual or any number of people at home, at work, or while walking through books, DVDs, or on the Internet and in various places. The establishment of this program has great social significance because it is likely to prevent lifestyle-related health issues such as cardiovascular diseases and increase healthy life expectancy.

There were some limitations to this study. First, participants might be more interested in laughter than usual because they applied for this study themselves, which may underestimate the effects of laughter. Further, as the missing values were substituted with the baseline values for the intention to treat analysis, it is possible that the laughter effect was underestimated. Second, it was difficult to separate the laughter effects from the exercise effects because as laughter also involves a type of exercise, it is impossible to separate the two. It was also difficult to separately evaluate the laughter programs effects from the laughter yoga (simulated laughter) and *rakugo* (spontaneous laughter). A synergistic effect could have been achieved by combining the two laughter types into the laughter program. Third, the molecular markers were not measured. However, laughing can reduce serum and salivary cortisol levels [29, 41], both of which are associated with obesity. The laughter program in this study improved subjective stress and subjective well-being. It is possible that the laughter program in this study had an aerobic effect, led to stress relief, reduced sympathetic nervous system tension, and affected the metabolic syndrome through the endocrine system, which includes the hypothalamic-pituitary-adrenal cortical system. Therefore, more detailed studies are needed to confirm these possible relationships fully. Fourth, there were minor differences in methods by area and the teachers giving the lectures or the laughter yoga. However, the main interventions were largely the same because the laughter yoga teachers were all taught by the same teacher, and we also adjusted the results by area. Fifth, it might be difficult to generalize the results of this study itself because of the small number of men in the sample; further research needs to be conducted after adjusting the schedule so that men can also participate.

The strength of this study is that we conducted a large, randomized intervention trial in a multi-center setting and had a high attendance rate. Previous intervention studies using laughter have been limited to a small number of participants and regions. In this study, however, the number of participants was 235 in an RCT, and the studies were conducted in Fukushima, Osaka, and Okayama, with similar effects obtained by area. On the basis of these results, there is a high possibility that this laughter program can be popularized and contribute to physical and mental health in the future. Furthermore, although health classes have been held in various municipalities, especially for the elderly, these classes’ low attendance and continuity have been seen as a problem [42]. In our study, there was a high likelihood that the class could be continued after the study, because the dropout rate of the laughter group was as low as 3.4%, the average attendance rate was high (84.8%), the class was enjoyable. There are laughter yoga circles across the country.

In conclusion, the comprehensive laughter program that combines simulated laughter (laughter yoga) with spontaneous laughter (*rakugo*) and lectures on laughter and health may help reduce body weight in predominantly elderly participants with metabolic syndrome risk factors by reducing stress and improving HRQOL and mental health, such as subjective well-being and optimism. In the future, it is necessary to further examine and evaluate this laughter program as a feasible option for residents.

## Supplementary Information

**Additional file 1: Supplementary Table S1.** Changes and intervention effects in physical and mental health scores except dropouts.

## Data Availability

The datasets generated and analyzed during the current study are not publicly available due to other researchers are currently partially analyzing, but are available from the corresponding author on reasonable request.

## Abbreviations

BMI: Body mass index
HRQOL: Health-related quality of life
LOT-R: Revised Life Orientation Test
PCS: Physical component summary
MCS: Mental component summary
RCT: Randomized controlled trial
SBP: Systolic blood pressure
DBP: Diastolic blood pressure
HbA1c: Hemoglobin A1c
SF-8: 8-Item Short-Form Health Survey
GDS-15-J: Geriatric Depression Scale 15
