# Supplementary material for: Effects of a laughter program on body weight and mental health among Japanese people with metabolic syndrome risk factors: a randomized controlled trial
Source: BMC Geriatr. 2022 Apr 23;22:361. doi: 10.1186/s12877-022-03038-y (PMC9035242; doi:10.1186/s12877-022-03038-y)
Supplement: Supplementary file 1 — Additional file 1: Supplementary Table S1. Changes and intervention effects in physical and mental health scores except dropouts. [file 12877_2022_3038_MOESM1_ESM.pdf]

Supplementary Table S1. Changes and intervention effects in physical and mental health scores except dropouts

Variables	Group	Examination [a]		Changes	Changes between laughter and control groups [b]	95% confidence intervals [c]	Multivariable-adjusted changes	*P* value [d]
		Baseline	12-week follow-up					
Number	Laughter	113	113					
	Control	112	112					
Body mass index, kg/m^2	Laughter	23.1 ± 3.5	22.8 ± 3.3	-0.28 ± 0.5	-0.19 ± 0.50	-0.32 – -0.06	-0.28	0.007
	Control	22.8 ± 3.2	22.7 ± 3.2	-0.09 ± 0.5			-0.10	
Body weight, kg	Laughter	56.4 ± 10.1	55.7 ± 9.9	-0.68 ± 1.2	-0.44 ± 1.21	-0.76 – -0.12	-0.67	0.009
	Control	55.8 ± 9.6	55.6 ± 9.4	-0.24 ± 1.2			-0.25	
Abdominal circumference, cm	Laughter	85.2 ± 10.1	85.1 ± 9.8	-0.09 ± 3.3	-0.01 ± 3.50	-0.93 – 0.91	-0.07	0.92
	Control	85.1 ± 10.1	85.0 ± 9.7	-0.08 ± 3.7			-0.11	
Subjective mental stress, score	Laughter	2.3 ± 0.8	2.1 ± 0.8	-0.19 ± 0.6	-0.25 ± 0.63	-0.41 – -0.08	-0.17	0.004
	Control	2.2 ± 0.8	2.2 ± 0.8	0.06 ± 0.6			0.05	
Subjective well-being, score	Laughter	7.6 ± 1.6	8.1 ± 1.3	0.42 ± 1.3	0.41 ± 1.23	0.08 – 0.73	0.44	0.002
	Control	7.6 ± 1.4	7.6 ± 1.5	0.01 ± 1.2			-0.01	
Geriatric depression scale 15, score	Laughter	3.7 ± 2.9	3.1 ± 2.7	-0.58 ± 2.0	-0.29 ± 2.16	-0.87 – 0.28	-0.64	0.12
	Control	4.0 ± 3.1	3.7 ± 3.1	-0.28 ± 2.3			-0.22	
The Revised Life Orientation Test, score	Laughter	13.5 ± 3.2	14.4 ± 3.2	0.96 ± 2.6	0.58 ± 2.63	-0.11 – 1.27	1.04	0.03
	Control	13.1 ± 3.1	13.4 ± 3.3	0.38 ± 2.7			0.31	
SF-8 Health Survey Scoring								
Physical component summary	Laughter	46.7 ± 7.7	48.8 ± 6.6	2.10 ± 7.8	1.94 ± 7.75	-0.10 – 3.98	2.17	0.02
	Control	46.5 ± 7.7	46.6 ± 8.4	0.11 ± 7.8			0.04	
Mental component summary	Laughter	48.9 ± 6.1	49.8 ± 6.6	0.94 ± 6.8	1.16 ± 6.45	-0.53 – 2.85	0.87	0.24
	Control	49.3 ± 6.6	49.2 ± 7.2	-0.11 ± 6.3			-0.04	
Physical functioning	Laughter	47.6 ± 7.9	49.4 ± 6.5	1.84 ± 8.0	2.31 ± 8.53	0.07 – 4.55	1.96	0.009
	Control	47.3 ± 8.2	46.8 ± 9.4	-0.47 ± 9.1			-0.59	
Role physical	Laughter	48.3 ± 7.8	49.8 ± 5.9	1.57 ± 7.2	2.26 ± 7.51	0.29 – 4.23	1.50	0.01
	Control	48.6 ± 6.8	47.9 ± 7.7	-0.69 ± 7.8			-0.61	
Bodily pain	Laughter	48.4 ± 8.0	50.7 ± 8.0	2.34 ± 9.3	1.71 ± 8.69	-0.57 – 4.00	2.40	0.06
	Control	48.4 ± 8.8	49.0 ± 8.7	0.63 ± 8.0			0.57	
General health perception	Laughter	48.5 ± 6.3	50.8 ± 5.9	2.30 ± 6.0	1.27 ± 6.17	-0.35 – 2.89	2.32	0.07
	Control	48.4 ± 7.1	49.4 ± 7.4	1.03 ± 6.3			1.00	
Vitality	Laughter	49.2 ± 5.9	50.7 ± 6.2	1.53 ± 6.4	1.43 ± 5.91	-0.12 – 2.99	1.47	0.06
	Control	49.6 ± 5.9	49.7 ± 6.3	0.09 ± 5.3			0.15	
Social functioning	Laughter	48.3 ± 8.2	49.6 ± 8.1	1.24 ± 9.0	0.94 ± 8.59	-1.32 – 3.19	1.18	0.39
	Control	48.6 ± 8.7	48.9 ± 8.0	0.30 ± 8.1			0.36	
Role emotional	Laughter	49.3 ± 6.2	50.4 ± 5.0	1.03 ± 6.6	1.61 ± 6.75	-0.16 – 3.39	1.07	0.03
	Control	49.3 ± 6.3	48.7 ± 7.8	-0.58 ± 6.9			-0.62	
Mental health	Laughter	48.9 ± 6.3	50.6 ± 6.3	1.66 ± 6.3	1.93 ± 6.25	0.28 – 3.57	1.57	0.03
	Control	49.4 ± 6.7	49.3 ± 6.8	-0.09 ± 6.4			0.00	

Data are represented as means ± standard deviation and mean change. *P* values indicate the intervention effects of changes in the intervention and non-intervention periods. Changes in each group are shown as the value of the 12-week follow-up examination minus the value of baseline examination. The intervention effects of the laughter program on variables were analyzed using an analysis of covariance adjusted by age, sex, metabolic syndrome risk factors, medication, and area, and each dependent variable value at baseline. [a] Values are mean ± standard deviation. [b] Baseline and 12-week follow-up changes between the laughter and control groups. [c] 95% confidence intervals between changes in baseline and 12-week follow-up of between the laughter and control groups. [d] *P* values for comparing the adjusted changes from baseline to 12-week follow-up in the laughter and control groups using an analysis of covariance adjusted by age, sex, medication and area, and each dependent variable value at baseline. SF-8: 8-Item Short Form Health Survey.
